# Optimising beamformer regions of interest analysis

**DOI:** 10.1016/j.neuroimage.2014.08.019

**Published:** 2014-11-15

**Authors:** Ashwini Oswal, Vladimir Litvak, Peter Brown, Mark Woolrich, Gareth Barnes

**Affiliations:** a Wellcome Trust Centre for Neuroimaging, UCL Institute of Neurology, 12 Queen Square, London, UK; b Nuffield Department of Clinical Neurosciences, John Radcliffe Hospital, Oxford, UK; c Oxford Centre for Human Brain Activity (OHBA), Oxford, UK

**Keywords:** Beamforming, Regions of interest, Bayesian PCA

## Abstract

Beamforming is a spatial filtering based source reconstruction method for EEG and MEG that allows the estimation of neuronal activity at a particular location within the brain. The computation of the location specific filter depends solely on an estimate of the data covariance matrix and on the forward model. Increasing the number of M/EEG sensors, increases the quantity of data required for accurate covariance matrix estimation. Often however we have a prior hypothesis about the site of, or the signal of interest. Here we show how this prior specification, in combination with optimal estimations of data dimensionality, can give enhanced beamformer performance for relatively short data segments. Specifically we show how temporal (Bayesian Principal Component Analysis) and spatial (lead field projection) methods can be combined to produce improvements in source estimation over and above employing the approaches individually.

## Highlights

• This paper concerns optimising beamformer analysis for anatomical ROIs.
• Channel reduction is performed using an ROI projection and Bayesian PCA.
• This improves covariance matrix estimation for a given data length.
• The proposed approach results in improvements in source estimation.

## Introduction

Beamforming is an adaptive spatial filter based method of estimating electrical activity in the human brain based on signals from an M/EEG sensor array. Typically per-location summary statistics of electrical change are used to provide three-dimensional images of brain function. The spatial filter corresponding to a particular brain region is determined based on knowledge of the lead field matrix and from an estimate of the data covariance matrix (Van Veen et al., 1997, Gross et al., 2001, Hillebrand et al., 2005, Brookes et al., 2008). Precise estimation of both the lead fields and the data covariance is therefore essential for accurate beamformer solutions.

This paper focuses around the accuracy of covariance matrix estimation which, perhaps counterintuitively, is inversely proportional to the number of channels (Brookes et al., 2008). The logic being that one needs more data to make an accurate estimate of the covariance between more channels. In fact it can be shown that doubling the number of M/EEG sensors necessitates that the number of data samples (alternatively the time-bandwidth product) is increased four-fold in order to maintain the same covariance matrix estimation error (see Brookes et al., 2008 for further details). This can become a problem when one is interested in relatively short duration or narrow band phenomena (for example the 0.5–1 sec beta rebound, see Pfurtscheller and Lopes da Silva, 1999). In this paper we consider the case in which we do not require whole-brain coverage from the MEG system, but rather have a specific region of interest in mind. This allows us to decrease the effective number of channels and thereby to make either more accurate estimates or estimates of the same accuracy but with less data.

A well tested channel reduction approach involves projection to a sub-space designed to optimally represent sources within a region of interest (ROI) (see Taulu et al., 2004, Ozkurt et al., 2006, Rodríguez-Rivera et al., 2006 for an overview). Often the ROI may be selected a priori based on the experimenter's prior knowledge about areas of task related activity. For example, in Rodríguez-Rivera et al. (2006), the projection is based on the eigenvectors of the source leadfields within an anatomical ROI, and the number of orthogonal components for the projection must be specified by the user. Importantly, this approach for channel reduction incorporates information from all channels and has been shown to produce more precise source estimates than approaches, involving sub-selecting channels based on either power or location (see Rodríguez-Rivera et al., 2006 for more details).

Given a reduced set of sensors (or linear sensor combinations) there remains however the question of whether there is sufficient data to make a reliable covariance matrix estimate. Recent work has shown how using Bayesian PCA one can make an estimate of the latent dimensionality (effective useful number of channels) (Woolrich et al., 2011). Projecting the data into this space and hence ending up with a reduced covariance matrix based on fewer channels is equivalent to optimally regularizing (or diagonally loading) the full covariance matrix.

In this work we propose a two-step procedure that unifies the approaches described above. Firstly, an ROI projection is used to reduce the effective number of channel components a-priori. This step is only based on the forward model. Secondly, Bayesian PCA is applied to further refine the dimensionality estimate based on covariance of the ROI-projected data. As both steps have the effect of reducing the effective number of channels the covariance estimate becomes more robust for the same amount of data.

We proceed by outlining and demonstrating the use of ROI projection and bPCA separately. And then go on to show how the combination of these steps improves the accuracy and resolution of beamforming estimates.

## Methods

### Spatial dimensionality reduction (ROI projection)

Details of this method can be found in Rodríguez-Rivera et al. (2006). In what follows however we will provide a brief overview of the general principles.

We formulate sensor level MEG activity, *x*, measured at N channels and T time points as follows:

(1) $$x=\sum_{l=1}^{L}H\left(\theta_{l}\right)m\left(\theta_{l}\right)+q$$

*H*(*θ_l_*) is an N × 3 lead field matrix representing the scaling of the projection of a unit amplitude dipole at location *θ_l_*, to N channels, in the x, y and z directions respectively. Additionally *m*(*θ_l_*) represents a 3 × T matrix of time courses in the x, y and z directions (in this paper we will use the MNI coordinate system) for a dipole located at *θ_l_*, where *l* = 1…*L*. Activity is summed over all sources before adding isotropic Gaussian white noise, *q* to the sensors.

The goal is to find a transformation, *U_r_* that minimises the error between the representation of the activity of sources, selected from a ROI, in the original data and in the projected data. Assuming that *U_r_* is an N × M matrix with orthonormal columns, where M < N, the projected data takes the form.

(2) $$x_{r}={U_{r}}^{t}x$$

The N × T matrix *x* has been transformed to an M × T matrix, *x_r_* corresponding to a reduction in the number of channels from N to M. Rodríguez-Rivera et al. (2006) show that *U_r_* can be computed from the singular value decomposition of the following symmetric matrix:

(3) $$\mathrm{US}U^{t}=\sum_{r=1}^{R}H\left(\theta_{r}\right)H{\left(\theta_{r}\right)}^{t}\text{.}$$

Accordingly, *U_r_* is set to the M columns of *U* corresponding to the M largest eigenvalues of *B* (the eigenvalues may be determined from the diagonal of *S*). This last formulation simply reduces to the approach for dimensionality reduction used in Friston et al. (2008) (in which case the ROI was defined by the space of lead fields on the cortical surface). In addition to dimensionality reduction of the data, a new leadfield set is computed for each brain location *θ_l_* (see Eq. (10)). The above formulation can be applied when no prior information is available about the dipole moment, or when the dipole moment is known a priori e.g. in the case of surface constrained orientations. As an example of this if we consider a source with known orientation along the x-axis, the projection matrix *U_r_* would be computed only from the first column of *H*(*θ_r_*).

An important issue with this approach is selecting the dimensionality M. This dimensionality determines the trade-off between the accuracy of the representation of the ROI and the spatial resolution of the resulting projection. In other words, increasing M leads to a more accurate representation of sources in the ROI, but this comes at the cost of also representing sources outside the ROI. Further insights into this trade off can be gained by considering the mean squared error of the linear transformation, which is represented as the sum of the N–M smallest eigenvalues (given by the diagonal elements in S in Eq. (3)), normalised by the sum of all eigenvalues.

(4) $$e{\left(M\right)}^{2}=\frac{\sum_{M}^{N}\lambda_{i}}{\sum_{i=1}^{N}\lambda_{i}}$$

Lower values of this error are obtained by minimising the difference between N and M and are associated with more accurate representations of sources in the ROI. A local measure of the ability of the transformation, *U_r_* to represent sources is gained by considering the ratio of the projected source and the original source energies at each spatial location, which mathematically corresponds to the following.

(5) $$F_{M}\left(\theta_{l}\right)=\frac{\mathrm{tr}\left({U_{r}}^{t}H\left(\theta_{l}\right)H{\left(\theta_{l}\right)}^{t}U_{r}\right)}{\mathrm{tr}\left(H\left(\theta_{l}\right)H{\left(\theta_{l}\right)}^{t}\right)}$$

**Fig. 1:**
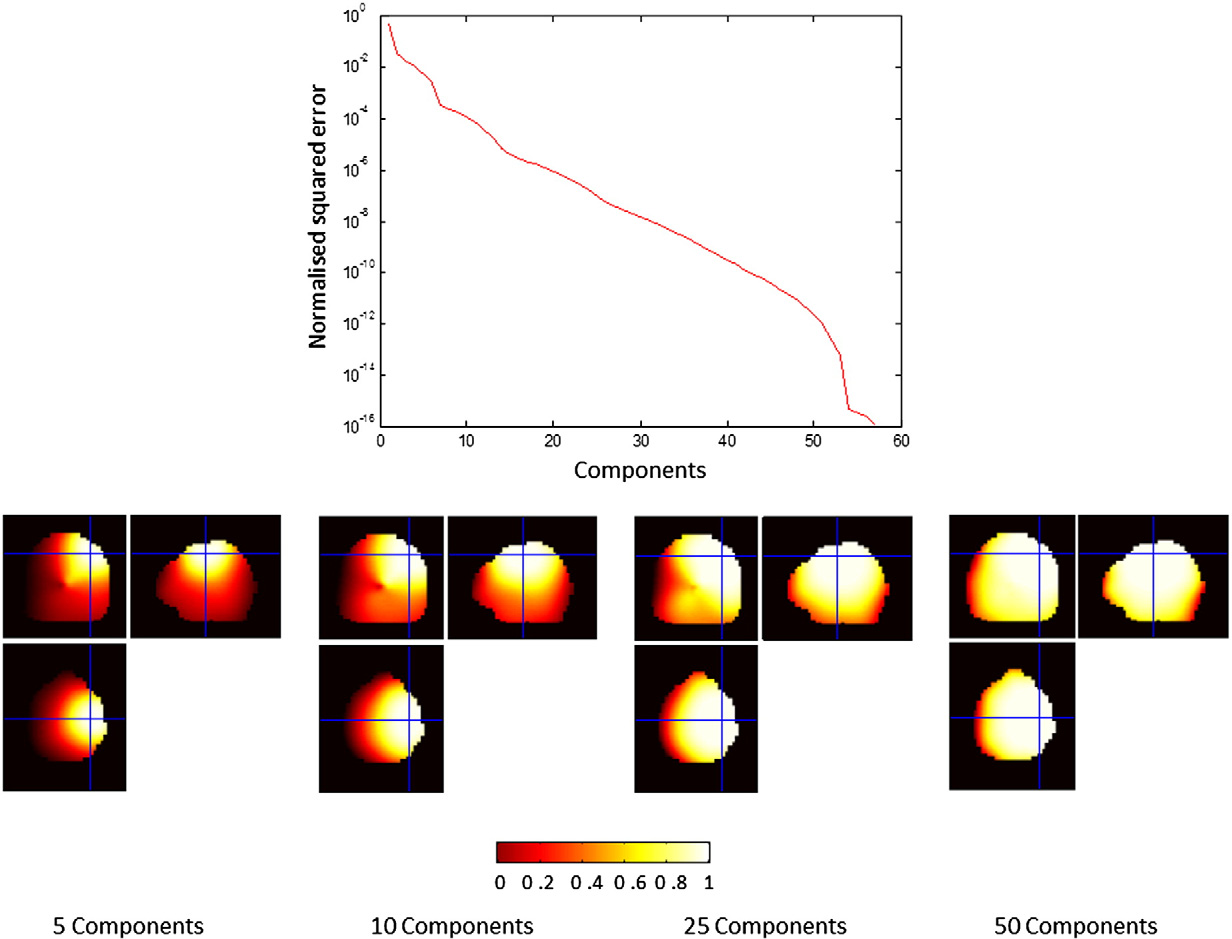
Features of the region of interest (ROI) projection method are displayed for a 1 cm^3^ cubic ROI centred at MNI co-ordinates 40–20 50 mm (the intersection of the cross-hairs). Source leadfields were computed at 5 mm intervals along the faces of the cubic ROI, giving a total of 27 leadfields from the ROI. The top panel displays how the normalised squared error varies with the number of principal components selected from the ROI (see Eq. (5) and also Methods). In the bottom panel of the figure, filtering functions, *F*_*M*_ representing the relative source energies in the projected and original data are computed for each 5 mm spaced grid point bounded by inner skull surface. The resulting data were linearly interpolated to create 2 mm resolution images (see Methods). The colourbar indicates the value of the filtering function, which is expressed as a ratio and therefore has no units. Filtering functions are displayed for different numbers of components selected from the ROI. It is evident that a trade-off exists between accuracy of the representation of the ROI and the spatial resolution of the resulting projection.

An ideal value for *F_M_*(*θ_l_*) is 1 for sources within the ROI and 0 for sources outside the ROI. From this it is evident that increasing M will increase the values of the numerator term for sources within and also outside the ROI (see Fig. 1). Additionally it is also evident that this term will depend on both the size of the ROI and on the sampling resolution of the leadfields within the ROI.

### Bayesian PCA (bPCA)

The underlying principle of Bayesian PCA is to estimate the true dimensionality of the data based on a generative model and appropriately selected priors. Within the context of beamforming, this estimated dimensionality is then used to act as a surrogate for finding the optimal amount of regularisation required to estimate the data covariance matrix. Expressed more formally, the generative model for bPCA is as follows:

(6) x=Gv+q.

Here the temporally demeaned data with dimensions N × T (see Eq. (2)) is represented by *x*. *G* is of dimensions N × P, where P corresponds to the principal component sensor maps. Finally, *v* is a P × T matrix of Gaussian latent (or hidden) variables which when multiplied by the principal component sensor maps with additive zero mean isotropic white noise, *q* ~ *N*(0, *σ*^2^*I*), result in the projected data. Woolrich et al. (2011) use a Variational Bayes (VB) approach (Bishop, 1999) based on Automatic Relevance Determination (ARD) hyperparameter thresholding in order to estimate the optimal number of components P, and hence the dimensionality of the data. An alternative approach based on Bayesian Model Selection (BMS) (Minka, 2008) has been shown to be both more accurate and also more computationally efficient, by virtue of avoiding an iterative VB updating routine. This is the approach we use in the present analysis.

The BMS approach involves computing the evidence for differing latent dimensionality models (or values of P from Eq. (6)) of the data. The model with the greatest evidence is then used to infer the true data dimensionality. In essence, a Gaussian likelihood function of the data given the PCA parameters is defined. Combining this likelihood with the required priors gives a complex integral for the model evidence that is efficiently and accurately approximated, using either Laplace's method or the Bayesian Information Criterion (BIC). In practice, the Laplace approximation tends to be more accurate and is for that reason used in the present paper. A detailed mathematical description and derivation of the BMS method can be found in Minka (2008), and a MATLAB implementation of the code is provided in the SPM12 distribution (see spm_pca_order.m).

In practice an SVD of the channel data *x* (or ROI projected data, see Eq. (12)) is performed yielding:

(7) $$U_{x}S_{x}V_{x}^{t}=x\text{.}$$

The bPCA approach selects the number of columns, P of *U_x_*, which are subsequently used to project the data to a lower dimensional subspace. Letting *U_p_* now represent the first P columns of *U_x_* the final projected data is represented by *x_p_* and has dimensionality P × T.

(8) $$x_{p}=U_{p}^{t}x$$

### Beamforming with combined ROI projection and bPCA

In this paper, depending on the approach used, the data for beamforming *x_b_* and leadfields for each brain location *H_b_*(*θ_l_*) take different forms and accordingly have different dimensions.

In the case of beamforming data without bPCA and the ROI projection:

(9) $$x_{b}=x\;\mathrm{and}\;H_{b}\left(\theta_{l}\right)=H\left(\theta_{l}\right)\text{.}$$

Here *x_b_* is an N × T matrix whilst *H_b_*(*θ_l_*) has dimensions N × 3. Similarly, in the case of just performing an ROI projection without bPCA, the data and leadfields take the following form where *x_b_* and *H_b_*(*θ_l_*) have dimensions M × T and M × 3 respectively (M < N, compare with Eq. (2)):

(10) $$x_{b}=x_{r}={U_{r}}^{t}x\;\mathrm{and}\;H_{b}\left(\theta_{l}\right)={U_{r}}^{t}H\left(\theta_{l}\right)\text{.}$$

When bPCA alone is performed the dimensions of *x_b_* and *H_b_*(*θ_l_*) are P × T and P × 3:

(11) $$x_{b}=x_{p}=U_{p}^{t}x\;\mathrm{and}\;H_{b}\left(\theta_{l}\right)={U_{p}}^{t}H\left(\theta_{l}\right)\text{.}$$

Finally, when the ROI projection is performed prior to bPCA, assuming that *U*_*p*_ is now calculated for *x*_*r*_ rather than *x* as per Eq. (7):

(12) $$x_{b}=U_{p}^{t}{U_{r}}^{t}x\;\mathrm{and}\;H_{b}\left(\theta_{l}\right)=\;{U_{p}}^{t}{U_{r}}^{t}H\left(\theta_{l}\right)\text{.}$$

Here the dimensions of *x*_*b*_ and *H*_*b*_(*θ*_*l*_) are L × T and L × 3, where L is less than M, N and P.

Linearly Constrained Minimal Variance (LCMV) beamforming (Van Veen et al., 1997) is a commonly used method of source estimation where the goal is to define a spatial filter, *W*(*θ*_*l*_) that when applied to the data, *x*_*b*_ gives an estimate of the source time course, $\hat{m}\left(\theta_{l}\right)$ (in the x, y and z directions) at a particular spatial location *θ*_*l*_:

(13) $$\hat{m}\left(\theta_{l}\right)=W\left(\theta_{l}\right)x_{b}$$

*W*(*θ*_*l*_) depends only on the data covariance matrix, C and the leadfield as follows:

(14) $$W\left(\theta_{l}\right)=\;\left(H_{b}^{t}(\theta_{l}\right)\;C^{-1}H_{b}{\left(\theta_{l})\right)}^{-1}H_{b}^{t}\left(\theta_{l}\right)C^{-1}$$

$$\mathrm{Where}\;C=\;\frac{1}{T-1}\sum_{t=1}^{T}\left(x_{b}\left(t\right)-\bar{x_{b}}\right){\left(x_{b}\left(t\right)-\bar{x_{b}}\right)}^{t}\text{.}$$

In this paper the covariance matrix C is estimated without additional regularisation.

### Validation with simulations

Simulated data were generated and analysed using MATLAB (Mathworks Inc., Natick, MA) with code from SPM12 (http://www.fil.ion.ucl.ac.uk/spm/), FieldTrip (http://fieldtrip.fcdonders.nl/) and SPM12 beamforming toolbox (https://code.google.com/p/spm-beamforming-toolbox/) (see also Litvak et al., 2011b, Oostenveld et al., 2011). Leadfields were computed for a 275 channel MEG system (CTF/VSM MedTech) using a single shell head model (Nolte et al., 2004), based on an inner skull mesh derived by inverse normalising a canonical mesh to a single subject's MRI scan (Mattout et al., 2007).

### Simulation 1

The aim of this simulation was to illustrate the complementary dimension reduction performance of ROI projection and the bPCA algorithm. We generated simulated data for 2 source configurations: 1) a 3 source simulation, with sources located at 10 mm spaced intervals (MNI co-ordinates 40–30 50, 40–20 50, and 40–10 50 respectively) and 2) a 27 source simulation with sources located at 10 mm spaced intervals on a cubic grid, centred on MNI co-ordinates 40–20 50. In each case, the orientations of the sources were selected randomly and each source had a Gaussian white noise time course. To maintain generality we did not explicitly fix the dipole moment to exist in the tangential plane and so these analyses should be regarded as conservative, with effective source moment less than or equal to that specified.

**Fig. 2:**
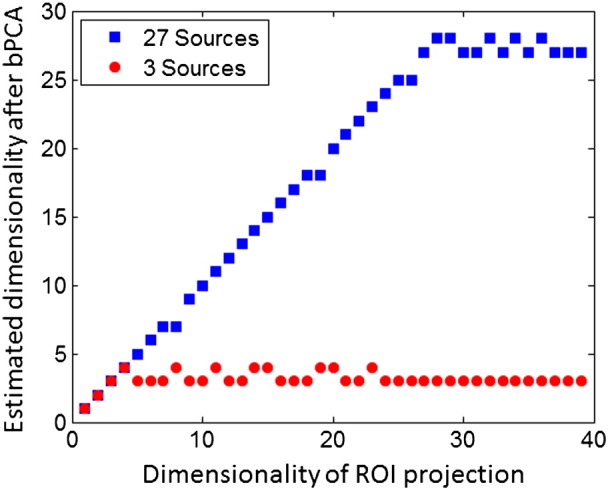
This figure highlights the results of simulation 1. Three and 27 sources were simulated as described in the Methods section and differing numbers of components were taken for the ROI projection (shown on the x axis) where the ROI comprised all sources. The dimensionality of the resulting projected data was consequently equal to the number of selected components. The y-axis highlights the dimensionality subsequently estimated after performing bPCA. The results for the 3 and 27 source simulations are shown by the red circles and blue squares respectively. Importantly, the use of bPCA following the ROI projection, results in accurate estimation of the number of sources in a ROI.

Ten seconds of MEG data were generated in total. Gaussian white noise was added to the simulated data such that the ratio of the RMS (root mean squared) amplitude of the signal to that of the noise was 5. Following the generation of simulated data a region of interest projection was performed, with the region of interest comprising all simulated sources. Crucially, we varied the number of components taken from the ROI, and applied a secondary bPCA step in order to determine the dimensionality of the resulting projected data (see Fig. 2 and Methods).

### Simulation 2

**Fig. 3:**
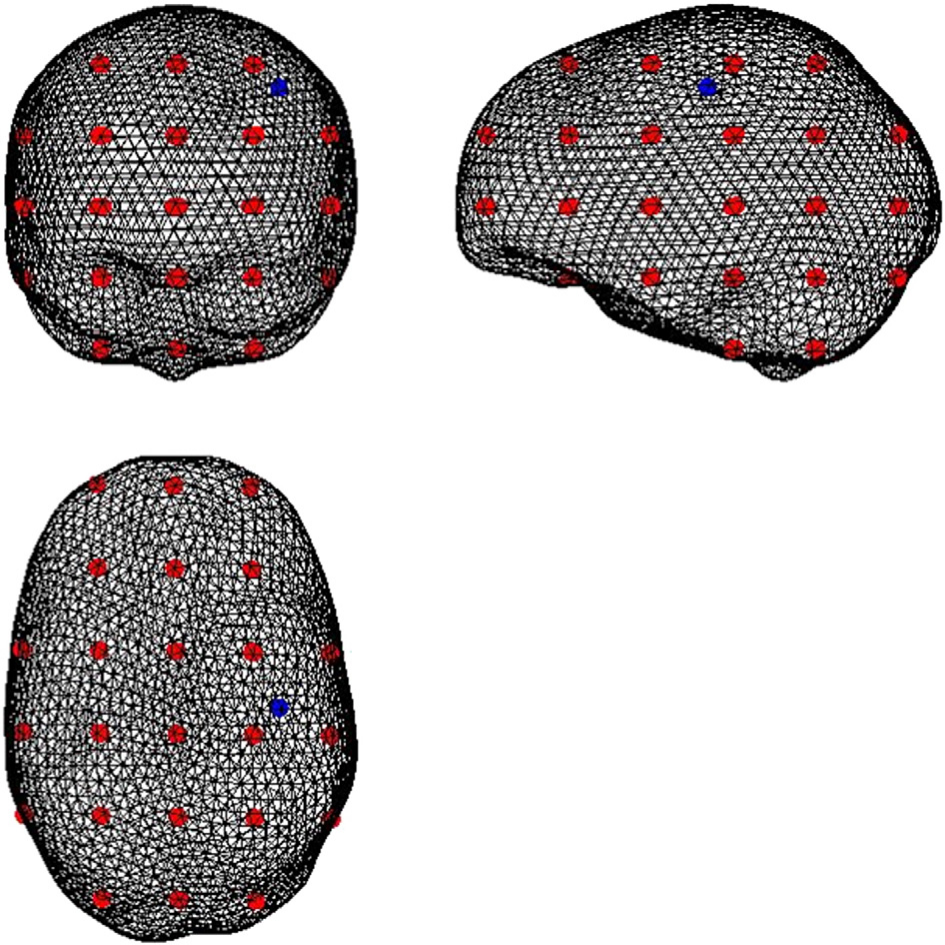
Here we display an illustration of the set-up of the second simulation. As per the first simulation a 20 Hz sinusoidal source shown in red, was simulated at MNI co-ordinates 40–20 50. 75 additional noisy sources shown in blue with Gaussian white noise time courses were positioned on 30 mm spaced grid points within the brain (see Methods for further details). Coronal, sagittal and axial sections through the image are shown. Comparisons of the three reconstruction methods for this simulation are shown in subsequent figures.

The aim of this simulation was to compare source localisation metrics in a realistic setting for three approaches of dimensionality reduction: 1) bPCA 2) the ROI projection and 3) a combination of the bPCA and the ROI projection. Background brain activity was represented by 75 randomly oriented sources, with independent Gaussian white noise time courses positioned on a 30 mm spaced grid within the brain as shown in red, in Fig. 3. An additional source with a sinusoidal 20 Hz time course (the source of interest), shown in blue in Fig. 3 was positioned at the right primary motor cortex (M1, MNI co-ordinates 40–20 50), to mimic motor cortical activity that might occur naturally during a movement task. This source was oriented along the tangential y direction (in MNI space). Gaussian white noise was added to the sensor signals such that the channel level signal-to-noise ratio (which we denote cSNR) – defined as the ratio of RMS amplitude of the signal originating within the brain to the RMS amplitude of the noise at MEG channels – was 5.

Ten trials of data of varying lengths were simulated, whilst also altering the ratio of the RMS amplitudes of the source of interest and the noisy sources. This allowed us to explore the effects of altering the source level signal-to-noise ratio (which we denote sSNR, in order to avoid confusion with cSNR) and also the time window for covariance matrix estimation. In order to make the simulation as realistic as possible, two experimental conditions were simulated, with half the trials belonging to each condition. The noisy brain sources were continuously active in both conditions, but the source of interest was active only in one of the conditions and it was inactive in the other. The conditions were thus labelled source ‘on’ and ‘off’ respectively, allowing us to also generate a t-statistic of the contrast between the two conditions. Ten realisations of simulated data were computed in order to determine standard errors.

Following the generation of simulated data, the data dimensionality was reduced using one of the three approaches described above. The ROI was a 1 cm^3^ cubic region surrounding the M1 source with 5 mm spatial sampling, yielding 27 leadfields. No additional regularisation was applied to the resulting data covariance matrices. LCMV beamforming was then used to reconstruct source power in the 10–40 Hz band for each trial of the two experimental conditions on a 5 mm grid in MNI space, bounded by the inner skull surface. For each grid point a two-sample *t*-test was then performed, contrasting the source ‘on’ and source ‘off’ conditions in order to yield a single t statistic. We compared the absolute value, localisation error in mm and spatial extent or FWHM (full width at half maximum) of the peak, closest to the source of interest, in the volumetric t statistic image between the different reduction methods. In order to ensure accurate localisation of the peak, beamformer estimates were also made on a finer 1 mm grid extending 5 mm around the ideal peak location. The FWHM was used as a measure of spatial resolution. It was calculated by fitting a Gaussian to the t-statistic profile computed at 1 mm intervals away from the peak along the y axis, and setting FWHM = $2\sqrt{\ln2}\sigma_{\mathrm{FWHM}}$, where *σ*_*FWHM*_ represents the standard deviation of the fit Gaussian. Finally, source timeseries were extracted at the location of the peak t statistic for the trials belonging to the source ‘on’ condition. The coefficient of determination (R^2^) was then computed between the simulated source of interest timeseries and the mean reconstructed timeseries across trials. We also computed the ratio of the mean RMS amplitude of the reconstructed source across trials to that of the simulated source. This provides insight into the degree with which simulated source power is recovered with the different methods.

Finally, we performed an additional simulation in order to gain additional insight into the spatial specificity of the three different approaches for the setup described above. The ROI was shifted in 5 mm intervals along a line in the x direction in MNI space towards the opposite hemisphere. The size of the ROI was 1 cm^3^ from which 27 lead field components were taken. We looked at the value of the peak t-statistic within each shifted ROI for three different conditions: 1) the ROI projection alone, 2) the ROI projection in addition to bPCA and 3) bPCA alone (in this case we were looking at different sections of a single beamformer image). As we expected the ROI techniques to excel for small amounts of data we explored two different trial lengths of 700 ms and 7000 ms whilst keeping the sSNR fixed at 0.

### Assessment of method on real data from a single patient with externalised DBS electrode

We demonstrate the application of dimensionality reduction methods on 180 s of resting data, epoched into 3 second long trials, collected from a Parkinsonian patient with bilateral therapeutic Deep Brain Stimulation (DBS) electrodes in the subthalamic nucleus (STN). The electrodes were externalised in the days following initial insertion facilitating simultaneous resting MEG and local field potential (LFP) recordings from the STN. A detailed discussion of the technical challenges of this type of recording and the pre-processing stages including description of the MEG artefacts caused by the percutaneous extension wires from the electrodes can be found in Litvak et al. (2010). Data from this patient has also been used in the following previous studies (Litvak et al., 2011a, Litvak et al., 2012, Oswal et al., 2013a, Oswal et al., 2013b).

As was previously shown (Litvak et al., 2010) the percutaneous ferromagnetic wires implanted under the patient's skin produce high-amplitude artefacts in the MEG signal. The topographies associated with these artefacts occupy the leading eigenvectors of the data. Consequently, application of the bPCA algorithm is unlikely to offer immunity to the large artefacts encountered. We show that projecting the data to a subspace spanned by the leadfields of an ROI prior to using bPCA provides better artefact suppression and hence greater statistical sensitivity.

We compared t images of beta band (15–30 Hz) coherence between the STN and whole brain for bPCA and the ROI projection followed by bPCA. Based on prior knowledge about increased beta band coupling between STN and primary motor cortex in Parkinson's disease (Hirschmann et al., 2011, Litvak et al., 2011a) the ROI was chosen to be an 8 cm^3^ cubic volume centred on the left primary motor cortex (M1). We used a slightly larger ROI in the case of patient analysis in order to account for uncertainties in the location of M1 due to head movements. Head movements may be more pronounced in Parkinsonian patients relative to healthy controls due to tremor or dystonia. We found that maximal head displacement was 1.64 cm and thus increased the edges of the cubic ROI from 1 to 2 cm. Leadfields were sampled at 10 mm intervals within the cubic ROI in order to construct the projection and 27 components were selected. In keeping with previous studies we used the Dynamic Imaging of Coherent Sources (DICS, Gross et al., 2001) beamforming approach to determine cortical sources coherent with a single bipolar left sided STN contact in the beta frequency band. This approach allowed us to generate volumetric images showing coherence between the STN and cortical regions. Although in previous studies we performed group level analysis of coherence images, in the present analysis we wished to determine the statistical significance of a single subject's resting STN-cortical coherence. We consequently generated 10 DICS images by randomly selecting trials of the epoched data with replacement (bootstrapped images) and further 10 DICS images (bootstrapped and shuffled images) where the coherence was computed after shuffling the STN data with respect to the channels. The shuffling served to destroy any physiological patterns of coherence, whilst any coherence induced by artefacts would be similar in the shuffled and bootstrapped images. A two-sample *t* test was subsequently performed in order to determine the spatial locations at which coherence in the bootstrapped images exceeded that in the shuffled images. This approach is similar to previously employed bootstrap approaches for single subject analysis in electrophysiology (Maris, 2012). Statistical analyses were performed in SPM12, and all reported findings are significant with family wise error correction at the peak level (p < 0.05).

## Results

### Spatial dimensionality reduction

Fig. 1 displays features of the ROI projection method for a 1 cm^3^ cubic ROI centred at MNI co-ordinates 40–20 50 mm. Specifically we highlight here the trade-off between the error of the representation of sources in the ROI and spatial selectivity of the ROI — as a function of the number of principal components taken from the ROI. The mean squared error of the ROI shown in upper panel of Fig. 1 was calculated as per Eq. (7), whilst the spatial filtering properties of the ROI were calculated as per Eq. (8) for grid points spaced at 5 mm intervals within the brain. This figure replicates the results shown in Rodríguez-Rivera et al. (2006). Note that as the number of components increases, the squared error of the representation of all sources in the ROI decreases, but the effective size of the ROI grows.

### Simulation 1

The results of simulation 1 are shown in Fig. 2. It is important to note in this case that the ROI comprised all active brain sources — either 3 sources or 27 in this case. In both cases, as per the findings of Woolrich et al. (2011), bPCA selects a dimensionality equivalent to the number of sources. Consequently if the number of components selected from the ROI projection is equal to the number of brain sources the resulting projection of the data is equivalent to the projection yielded by the bPCA approach. Here we specifically wanted to ask whether the addition of bPCA to the ROI projection algorithm would allow us to recover the true dimensionality of the data in the case that more components were selected from the ROI than the number of active brain sources. Fig. 2 shows for both the 3 and 27 source simulations (shown by the red circles and blue squares) that the combination of the ROI projection and the bPCA method accurately recovers the true data dimensionality. For example, in the 3 source simulation, taking 3 or more components from the ROI projection and then applying bPCA consistently yields a dimensionality of between 3 and 4. Similarly in the 27 source simulation, taking more than 27 components from the ROI and then applying bPCA consistently yields a dimensionality of between 27 and 28. In both cases, the errors are well within the accuracy of the bPCA algorithm we used (Minka, 2008 for further details). Note in these figures we have also established the behaviour of applying bPCA to the ROI projection when fewer components are taken from the ROI than the number of active brain sources. In this case, applying bPCA does not produce any additional decrement in the estimated dimensionality.

### Simulation 2

We have demonstrated in simulation 1 that the combination of the ROI projection and bPCA can accurately recover the dimensionality of an ROI. The goal of simulation 2 was to compare bPCA with the ROI projection for a realistic scenario and to determine whether the use of the ROI projection prior to bPCA could prove beneficial in terms of suppressing brain activity outside of the ROI.

**Fig. 4:**
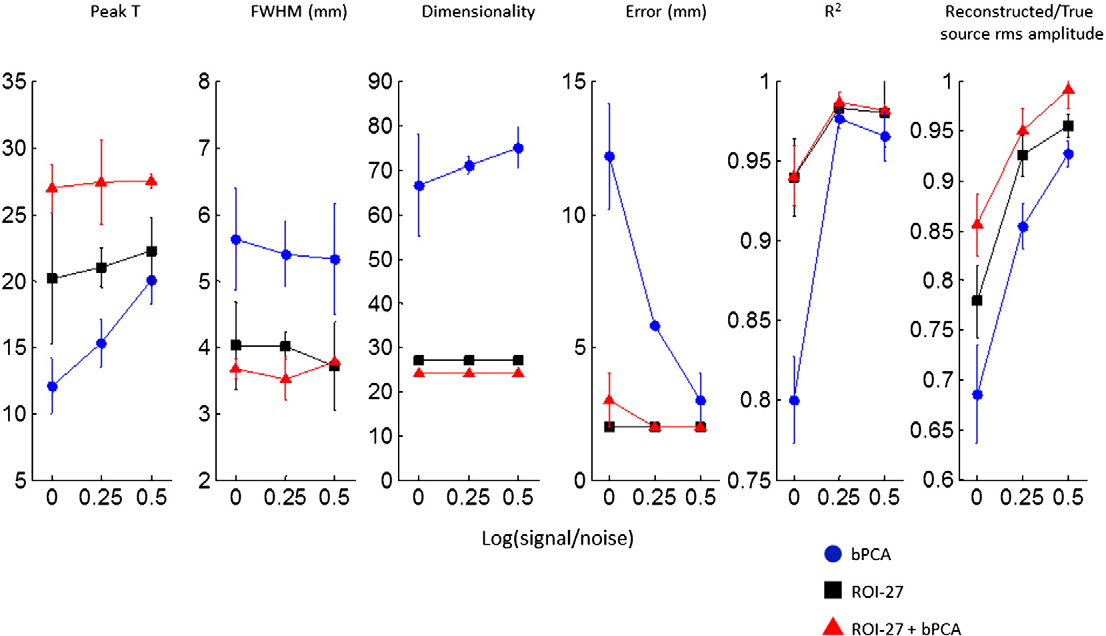
Results of simulation 2: Bayesian PCA (bPCA) is compared with an ROI projection with 27 components (ROI-27) and combination of the two approaches (ROI-27 + bPCA) for the set up described in the methods and shown in Fig. 3. The three approaches are compared for different SNRs of the source of interest in terms of: 1) The peak t value closest to the source of interest 2) The associated FWHM 3) The R^2^ correlation coefficient between the simulated and reconstructed source of interest timeseries for the ‘on’ condition 4) The source localisation error and 5) The ratio of the reconstructed to the simulated RMS amplitude of the source of interest for the ‘on’ condition. The final data dimensionality is also displayed for each approach. The error bars represent standard errors for the 10 realisations of simulated data.

Fig. 3 illustrates the position of noisy brain sources (shown in red) and a source of interest (shown in blue) that a 1 cm^3^ ROI was constructed around. Fig. 4 shows how source localisation measures specifically for the source of interest vary for bPCA, the ROI projection and the ROI projection plus bPCA as a function of the SNR of the source of interest compared to the noisy brain sources. In all cases a fixed trial length of 1000 ms was used. In addition to showing the improvement in source estimation with increasing SNR for all three approaches, this figure highlights that across a range of SNRs (and particularly at low SNRs) the ROI projection performs better than bPCA (as bPCA has no prior information on the source of interest). More specifically, peak t values are increased and their full width half maxima are reduced. Furthermore, the absolute error in source localisation is reduced and the R^2^ correlation coefficient between the simulated and reconstructed source of interest time course is increased. The increase in the R^2^ is most notable at low SNR levels. The estimation of the true source amplitude is also improved.

Interestingly, combining the ROI projection with bPCA produces some improvements over the ROI method alone — namely a greater peak t statistic, improved estimation of source amplitude and a reduction in the FWHM. The absolute localisation error and the R^2^ correlation coefficient were not markedly improved by the addition of bPCA to the ROI approach. The estimate of data dimensionality from the three algorithms is also shown in the figure for comparison. As per Woolrich et al. (2011) bPCA adapts the estimated data dimensionality for a given sSNR and time window for covariance matrix estimation. The dimensionality estimate for both the ROI projection and the ROI projection plus bPCA approaches is, however, more stable across the range of trial lengths and sSNR levels.

**Fig. 5:**
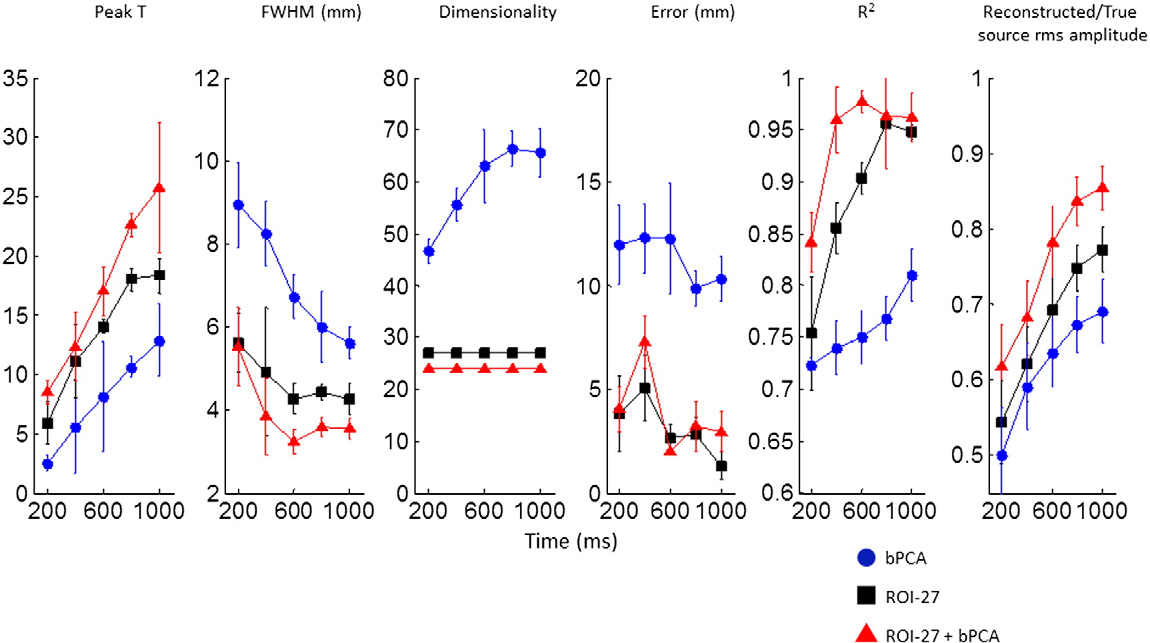
Results of simulation 2: Bayesian PCA (bPCA) is compared with a ROI projection with 27 components (ROI-27) and combination of the two approaches (ROI-27 + bPCA) for the set up described in the Methods and shown in Fig. 3. This time however, the trial length for covariance matrix estimation is varied for a fixed SNR level of 0. As per Fig. 4, the three source localisation approaches are compared in terms of the peak t statistic, the FWHM, the R^2^ correlation coefficient, the source localisation error and the extent to which the true source RMS amplitude is recovered. Final data dimensionalities are once again displayed and the error bars represent standard errors for the 10 realisations of simulated data.

We next explored the effect of altering the time window for covariance matrix estimation. The results for a fixed log sSNR of 0 (equal amplitude of signal and noise sources) are shown in Fig. 5. Ten trials of varying lengths were simulated as described in the Methods section. Once again we see that the ROI projection produces benefits over bPCA in terms of source localisation metrics across a range of trial lengths. This finding is in keeping with the concept that reducing the data dimensionality in order to represent an ROI reduces the number of samples of data required to accurately estimate the covariance matrix for beamforming. Furthermore, as before adding bPCA to the ROI projection produced improvements in the peak t statistic, source amplitude estimates, and the FWHM relative to the ROI projection alone. Additionally the R^2^ correlation coefficient was also improved by combining the ROI projection with bPCA at moderate trial lengths.

**Fig. 6:**
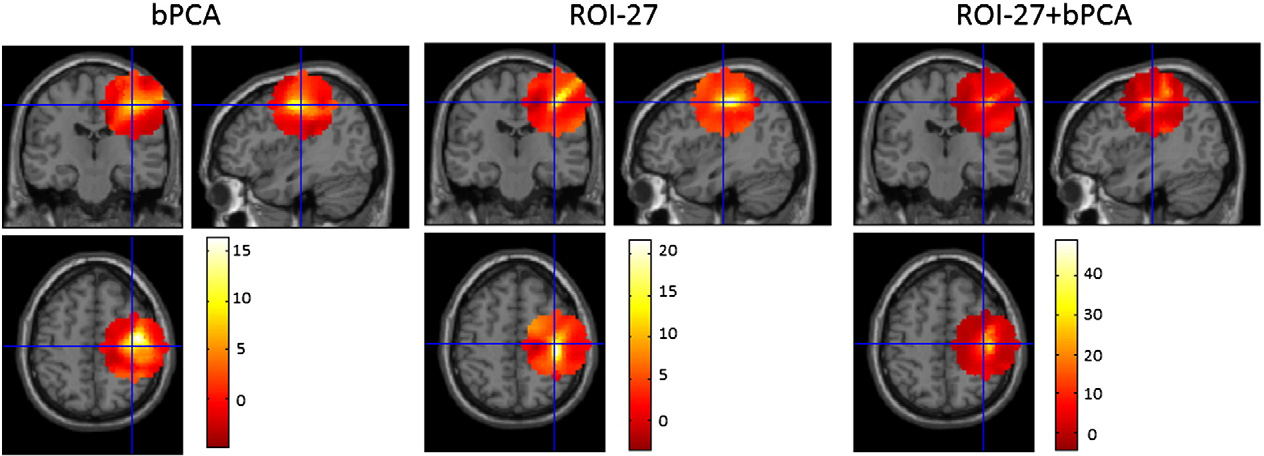
T-images contrasting the source ‘on’ and source ‘off’ conditions, superimposed on a canonical T1-weighted MRI, for a 30 mm spherical volume surrounding the source of interest (at MNI coordinates 40–20 50) are displayed. Coronal, sagittal and axial sections through the images are shown with the crosshairs centred on the source of interest. Note that colour bars are shown separately for each image. Combining the ROI projection with bPCA results in the highest t values in the vicinity of the source of interest.

These data highlight that significant improvements in source estimation can be produced by dimensionality reduction designed to optimally represent sources in an ROI. In Fig. 6, example t images of the three reconstruction approaches for a trial length of 1000 ms and a log SNR of 0 superimposed on a canonical T1-weighted MRI are displayed. This figure displays a spherical volume of 30 mm radius surrounding the source of interest and again shows that enhanced and more focal t statistics can be produced by the ROI projection and its combination with bPCA.

**Fig. 7:**
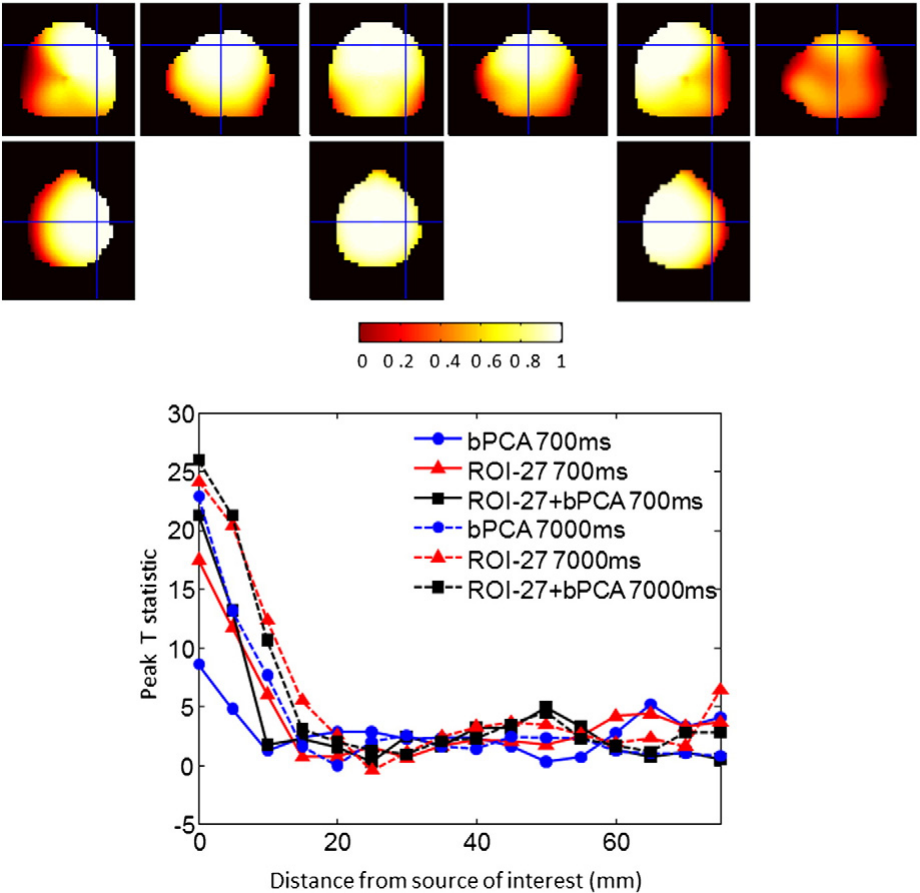
In the upper panel, three spatial filtering profiles are displayed, as per Fig. 1, for a 1 cm^3^ ROI with 27 components centred on the following MNI co-ordinates: − 40–20 50, 0–20 50, 40–20 50. The cross-hairs are centred on the source of interest located at 40–20 50. The colourbar indicates the value of the filtering function, which is expressed as a ratio and therefore has no units. The bottom half of the figure shows the value of the peak t statistic within a 1 cm^3^ ROI, as the ROI is shifted distances (in the x direction) from the source of interest. The results are displayed for two different trial lengths, for: 1) the ROI projection with 27 components (ROI-27), 2) bPCA (i.e. just a single optimally regularised beamformer) and 3) the ROI projection followed by bPCA (ROI-27 + bPCA). Note that the main differences between the three approaches are for the small amount of data (solid lines); in this case the ROI + bPCA (solid squares) performs as well as a beamformer with ten times the amount of data (dotted circles).

Fig. 7 highlights the spatial specificity of the three different reconstruction approaches for long and short time windows for covariance matrix estimation. The top panel shows spatial filtering functions (as per Fig. 1) centred on MNI co-ordinates 40–20 50, 0–20 50 and − 40–20 50 for illustration. The bottom panel displays the profile of the peak t statistic within each 5 mm shifted ROI. The results are displayed for two different trial lengths for each of the three different reconstruction approaches. For large amounts of data (7000 ms) the covariance matrix estimate is precise and ROI manipulations do little to enhance beamformer performance (compare ROI and ROI + bPCA to bPCA—a single optimally regularised beamformer); indeed although there is a slight increase in the peak t-statistic at the source itself, the spatial specificity (the fall off with distance) degrades marginally due to fewer effective channels. For small amounts of data however (700 ms) the ROI based approaches make maximal use of the data available and considerably improve on the single beamformer image (bPCA) with ROI + bPCA performing at the level of an optimally regularised beamformer with ten times the data.

### Patient data

**Fig. 8:**
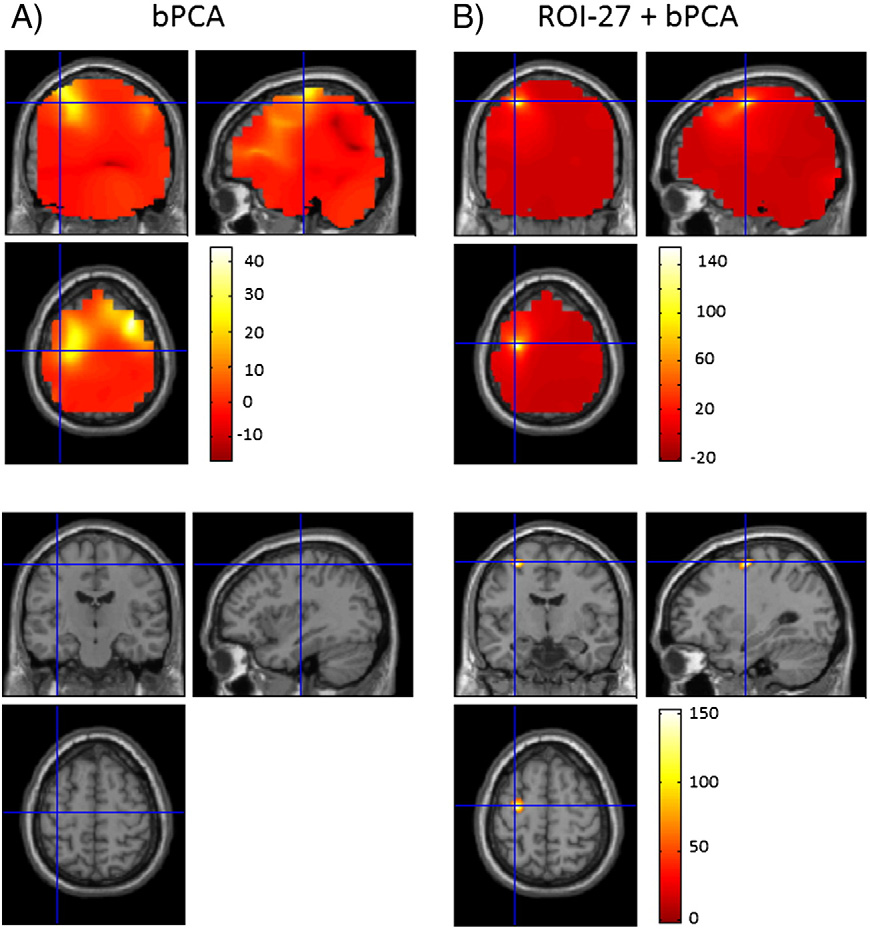
The results of analysis of patient data are displayed. The upper halves of panels A and B display t images of coherence between the left STN and cortical areas, computed using bPCA and the ROI projection (with 27 components) combined with bPCA. The ROI was selected as a cubic volume surrounding M1 (see Methods section for more details). T images are superimposed on a canonical T1-weighted MRI. Combining the ROI projection with bPCA results in more focal t images and a greater peak T statistic (indicated by the colour bars). In the case of bPCA, no voxels survived family wise error (FWE) correction at the peak level (p < 0.05) as shown by the lack of highlighted voxels in the lower half of panel A. When the ROI projection was combined bPCA however, 120 voxels survived FWE correction at the peak level, indicated in the lower half of panel B. The cross hairs in panels A and B are centred as described in the Methods section.

Fig. 8 highlights the results of the analysis on patient data. In the upper panels we display t statistic images of coherence between the left STN and whole brain areas for bPCA and the ROI projection followed by bPCA with 27 components selected from the ROI. In the case of bPCA, the crosshairs are located at the peak closest to M1, at MNI coordinates − 36–16 58. In this case, bPCA selected a data dimensionality of 141. Importantly, no voxels survived family wise error correction at the peak level (p < 0.05). Combining the ROI projection with bPCA produced more focal images and also resulted in a greater peak T statistic as indicated by the colour bars in Fig. 8 panel B. Furthermore, voxels surviving statistical comparison at the peak level and are indicated in the lower half of panel B. The crosshairs in panel B are located at the peak closest to M1 at MNI coordinates − 30–8 60.

The patient data provide empirical evidence that combining the ROI projection with bPCA can provide increased statistical sensitivity in the presence of large artefacts and a low data SNR.

## Discussion

In this paper we have presented a framework for ROI analysis using beamformers. The choice of an anatomical ROI prior to source estimation has a number of benefits. In addition to excluding uninteresting variance or noise from other regions the brain (or environment), it reduces the effective signal space (decreasing the effective number of channels) thereby improving the estimation of the data covariance. This translates in practice to better artefact immunity and more accurate source reconstruction as quantified by lower FWHM and higher accuracy of the reconstructed time-series.

Importantly, the use of Bayesian PCA to estimate dimensionality removes any arbitrary regularisation stage and can in principle accurately select the number of components from an ROI (Fig. 2). Additionally, projection of the data to an orthogonal subspace prior to bPCA is desirable, since bPCA subsequently selects orthogonal components in the data.

We think these methods will have specific application in the analysis of non-stationary MEG data where time is short and spatial hypotheses are well defined. For example, recent work (Woolrich et al., 2013, Baker et al., 2014, Brookes et al., 2014) has shown that resting state dynamics display distinct spatio-temporal modes such that different sections of the sensori-motor cortex coordinate within different time-windows. The pre-selection of this ROI (e.g. sensori-motor cortex) *a priori* would mean that a more robust characterisation of such modes would be possible within relatively short time windows. We should also note that there is no reason why the ROI needs to be spatially contiguous — the leadfields of a number of spatially distinct ROIs could be combined (see Eq. (3)) in order to yield a single linear transformation that effectively encompasses multiple regions of interest.

Although our results are encouraging we stress a number of practical issues. The question of how many leadfield components to select from the ROI is one that needs to be answered by the experimenter, but it can be appropriately selected based on the kinds of filter response profiles generated in Fig. 1 whilst bearing in mind that the selected number will limit the maximal number of orthogonal modes in the data (i.e. an upper limit for bPCA, see Fig. 3). Additionally the size of the ROI needs to be specified, but this can be based on a prior hypothesis about the spatial extent of task related activations — for example one may want to sample the entire the entire visual cortex in the case of visual paradigms. There are a number of ways in which this method could be compromised. The most obvious perhaps is that the selection of an ROI where no signal exists could lead to erroneous inference. On similar note, we have not explicitly examined the effect of correlated sources and how these would interact (if one were slightly outside the ROI but not completely suppressed).

It is also important to note that bPCA relies on a generative model of the data that assumes data samples to be independent and drawn from a Gaussian distribution. Such a model is not guaranteed to be appropriate for realistic experimental data, and in these cases directly estimating the data distribution via kernel density based approaches may provide more accurate solutions (see Mohseni et al., 2013 for detailed discussion). Another issue is that the signal to noise gain by using the subspace methods may push the beamformer resolution beyond the grid resolution (Barnes et al., 2004) causing signal peaks to apparently disappear (as they become under sampled). It may therefore be necessary to reduce grid spacing in certain circumstances.

Finally, as shown in Fig. 7, these methods provide a way of balancing the spatial specificity of the beamformer image with the amount of data available. For large stationary data sets with no artefacts one would expect very precise covariance matrices and correspondingly high spatial resolution of optimally regularised beamformer images which would hardly benefit from the ROI projections described here. In cases where the amount of stationary data is small, or in cases where artefacts (or correlated sources) exist in another region of the source space, we expect these methods to be of considerable benefit. For example, Fig. 7 shows that one can achieve the same spatial specificity with ten times less data using the methods we describe. We believe that these methods may also be of benefit in certain clinical studies or in studies with children where participants are unable to sit still for long periods of time and hence scanning durations are necessarily limited.

## References

[bb0005] Baker A.P., Brookes M.J., Rezek I.A., Smith S.M., Behrens T., Probert Smith P.J., Woolrich M. (2014). Fast transient networks in spontaneous human brain activity. Elife.

[bb0010] Barnes G.R., Hillebrand A., Fawcett I.P., Singh K.D. (2004). Realistic spatial sampling for MEG beamformer images. Hum. Brain Mapp..

[bb0015] Bishop C. (1999). Artif Neural Networks.

[bb0025] Brookes M.J., Vrba J., Robinson S.E., Stevenson C.M., Peters A.M., Barnes G.R., Hillebrand A., Morris P.G. (2008). Optimising experimental design for MEG beamformer imaging. NeuroImage.

[bb0020] Brookes M.J., O'Neill G.C., Hall E.L., Woolrich M.W., Baker A., Palazzo-Corner S., Robson S.E., Morris P.G., Barnes G.R. (2014). Measuring temporal, spectral and spatial changes in electrophysiological brain network connectivity. NeuroImage.

[bb0030] Friston K., Harrison L., Daunizeau J., Kiebel S., Phillips C., Trujillo-Barreto N., Henson R., Flandin G., Mattout J. (2008). Multiple sparse priors for the M/EEG inverse problem. NeuroImage.

[bb0035] Gross J., Kujala J., Hamalainen M., Timmermann L., Schnitzler A., Salmelin R. (2001). Dynamic imaging of coherent sources: studying neural interactions in the human brain. Proc. Natl. Acad. Sci. U. S. A..

[bb0040] Hillebrand A., Singh K.D., Holliday I.E., Furlong P.L., Barnes G.R. (2005). A new approach to neuroimaging with magnetoencephalography. Hum. Brain Mapp..

[bb0045] Hirschmann J., Özkurt T.E., Butz M., Homburger M., Elben S., Hartmann C.J., Vesper J., Wojtecki L., Schnitzler A. (2011). Distinct oscillatory STN-cortical loops revealed by simultaneous MEG and local field potential recordings in patients with Parkinson's disease. NeuroImage.

[bb0055] Litvak V., Eusebio A., Jha A., Oostenveld R., Barnes G.R., Penny W.D., Zrinzo L., Hariz M.I., Limousin P., Friston K.J., Brown P. (2010). Optimized beamforming for simultaneous MEG and intracranial local field potential recordings in deep brain stimulation patients. NeuroImage.

[bb0060] Litvak V., Jha A., Eusebio A., Oostenveld R., Foltynie T., Limousin P., Zrinzo L., Hariz M.I., Friston K., Brown P. (2011). Resting oscillatory cortico-subthalamic connectivity in patients with Parkinson's disease. Brain.

[bb0065] Litvak V., Mattout J., Kiebel S., Phillips C., Henson R., Kilner J., Barnes G., Oostenveld R., Daunizeau J., Flandin G., Penny W., Friston K. (2011). EEG and MEG data analysis in SPM8. Comput. Intell. Neurosci..

[bb0050] Litvak V., Eusebio A., Jha A., Oostenveld R., Barnes G., Foltynie T., Limousin P., Zrinzo L., Hariz M.I., Friston K., Brown P. (2012). Movement-related changes in local and long-range synchronization in Parkinson's disease revealed by simultaneous magnetoencephalography and intracranial recordings. J. Neurosci..

[bb0070] Maris E. (2012). Statistical testing in electrophysiological studies. Psychophysiology.

[bb0075] Mattout J., Henson R.N., Friston K.J. (2007). Canonical source reconstruction for MEG. Comput. Intell. Neurosci..

[bb0080] Minka T. (2008). MIT Media Lab Percept Comput Sect Tech Rep No 514.

[bb0085] Mohseni H.R., Kringelbach M.L., Woolrich M.W., Baker A., Aziz T.Z., Probert-Smith P. (2013). Non-Gaussian probabilistic MEG source localisation based on kernel density estimation. NeuroImage.

[bb0090] Nolte G., Bai O., Wheaton L., Mari Z., Vorbach S., Hallett M. (2004). Identifying true brain interaction from EEG data using the imaginary part of coherency. Clin. Neurophysiol..

[bb0095] Oostenveld R., Fries P., Maris E., Schoffelen J.-M. (2011). FieldTrip: open source software for advanced analysis of MEG, EEG, and invasive electrophysiological data. Comput. Intell. Neurosci..

[bb0100] Oswal A., Brown P., Litvak V. (2013). Movement related dynamics of subthalamo-cortical alpha connectivity in Parkinson's disease. NeuroImage.

[bb0105] Oswal A., Brown P., Litvak V. (2013). Synchronized neural oscillations and the pathophysiology of Parkinson's disease. Curr. Opin. Neurol..

[bb0110] Ozkurt T.E., Sun M., Sclabassi R.J. (2006). Beamspace magnetoencephalographic signal decomposition in spherical harmonics domain. Conf. Proc. IEEE Eng. Med. Biol. Soc..

[bb0115] Pfurtscheller G., Lopes da Silva F.H. (1999). Event-related EEG/MEG synchronisation and desynchronisation: basic principles. Clin. Neurophysiol..

[bb0120] Rodríguez-Rivera A., Baryshnikov B.V., Van Veen B.D., Wakai R.T. (2006). MEG and EEG source localization in beamspace. IEEE Trans. Biomed. Eng..

[bb0125] Taulu S., Kajola M., Simola J. (2004). Suppression of interference and artifacts by the signal space separation method. Brain Topogr..

[bb0130] Van Veen B.D., van Drongelen W., Yuchtman M., Suzuki A. (1997). Localization of brain electrical activity via linearly constrained minimum variance spatial filtering. IEEE Trans. Biomed. Eng..

[bb0135] Woolrich M., Hunt L., Groves A., Barnes G. (2011). MEG beamforming using Bayesian PCA for adaptive data covariance matrix regularization. NeuroImage.

[bb0140] Woolrich M.W., Baker A., Luckhoo H., Mohseni H., Barnes G., Brookes M., Rezek I. (2013). Dynamic state allocation for MEG source reconstruction. NeuroImage.

